# Unimpaired outcomes in 18-month-old borderline viable twins born at 22 weeks: A case report

**DOI:** 10.1097/MD.0000000000032571

**Published:** 2023-01-13

**Authors:** Wafaa Alrawi, Taisser Atrak, Ashraf Abuobayda, Nabil Elmansoury, Omar Elnakeib, Abhijeet Lonikar

**Affiliations:** a Department of Pediatrics, Sheikh Shakhbout Medical City, Abu Dhabi, United Arab Emirates; b SSMC Hospital in partnership with Mayo Clinic; and Khalifa University, Abu Dhabi, United Arab Emirates.

**Keywords:** 22 weeks, active intervention, case report, intact survival, periviable birth, preterm twins, extremely low birth weight, individualized management, periviable birth, survive

## Abstract

A gestational age of 22 to 23 weeks is the lower threshold for selective active intervention. Few infants delivered at a gestational age of 22 to 23 weeks survive if resuscitated. Among those who survive, most develop severe complications, especially in cases of multiple births at the limit of viability.

**Patient concerns:**

We report the intact survival of extremely preterm twins, a girl (Twin A) and a boy (Twin B), weighing 504g and 475g, respectively, born at the edge of viability at 22 2/7 weeks gestation without significant morbidity.

**Diagnoses:**

extremely preterm twins born at the edge of viability at 22 2/7 weeks.

**Interventions:**

Twin A required 6 weeks of mechanical ventilation. She received conventional and high-frequency oscillation ventilation. She was extubated to noninvasive positive airway pressure ventilation at 28 weeks and 2 days post conception. Twin B required longer duration of invasive ventilation lasting 11 weeks. Moreover, he had several episodes of feeding intolerance and abdominal distension. However, his serial abdominal radiographs showed nonspecific findings. The gastric tubes were eventually removed from both twins. Full oral feeding was successful on discharge.

**Outcomes:**

Both infants are presently in good condition.They were discharged home with a full oral feeding, and without any respiratory support. Now they are 18-month-old with unimpaired development.

**Lessons:**

This report would support healthcare providers in decision-making. It highlights the importance of perinatal and neonatal management optimization to improve survival rates and clinical outcomes of periviable birth. In addition it emphasize the individuality of each case and the need to consider the parents’ wishes in the management decision.

## 1. Introduction

The gestational limit of survival, commonly established as a gestational age of 22 to 25 weeks, has decreased over the past few years due to technological advances in neonatal and obstetric care. Consequently, the survival rates of significantly premature babies have improved; yet, their morbidity rate has increased.^[1–3]^

Recent data have shown continuous improvements in general health and neurodevelopmental outcomes and an increasing tendency to consider active interventions for infants at the edge of viability.^[4,5]^There are several reported cases of singleton infants delivered before 23 weeks of gestation who survived without significant disabilities and achieved appropriate neurodevelopment.^[6,7]^ The intact survival of twins born before 23 weeks are rare.

## 2. Case report

A 30-year-old woman who was pregnant with diamniotic dichorionic twins at 22 2/7 weeks of gestation was admitted to the delivery room for preterm labor. She experienced bleeding, and her membranes had ruptured 23 hours before admission. Her condition was complicated by chorioamnionitis, diagnosed due to fever, tachycardia, and elevated inflammatory markers. She had a white blood cell count of 24 × 10^9^/L, an absolute neutrophil count of 23 × 10^9^/L, a C-reactive protein level of 105 mg/L, and a negative test result for procalcitonin. Both fetuses exhibited good cardiac activities and active movements. The mother had conceived following in vitro fertilization and embryonal transfer. She was also the mother of healthy 3-year-old twins, who were also conceived by in vitro fertilization. The medical team offered the parents comfort and counseling regarding proactive care, intensive care, and predicted outcomes. The parents requested that all available life-support measures be initiated, despite their complete understanding of the risks and long-term expected problems.

The parents’ decision to initiate full resuscitation was unique to our unit. A complete plan, with some flexibility under our policy, was adopted in consultation with the obstetricians and parents. After completing the prenatal steroid course, a live female infant (Twin A) weighing 504 g with Apgar scores of 4, 5, and 7 at 1, 5, and 10 minutes, respectively, was spontaneously delivered. Subsequently, a live male infant (Twin B), weighing 475 g, with breech presentation and Apgar scores of 3, 5, and 7 at 1, 5, and 10 minutes, respectively, was delivered. Both newborns unexpectedly were born in good condition. Each of them was resuscitated, intubated, and administered 1 dose of surfactant.

Twin A required 6 weeks of mechanical ventilation. She initially received conventional respiratory support; however, within a few days, she required high-frequency oscillation. Thereafter, she was extubated to noninvasive positive airway pressure ventilation at 28 weeks and 2 days post-conception and, eventually, to nasal high-flow oxygen. Two weeks later, Twin B was extubated to biphasic positive airway pressure. However, after a few days, he required reintubation and invasive ventilation. His second intubation lasted for 3 weeks because of bilateral irreducible inguinal hernias that required urgent herniotomy. He was finally successfully extubated after 11 weeks of invasive ventilation. He then received continuous positive airway pressure on day 92 (i.e., 33 weeks and 3 days post-conception). The patient successfully weaned to nasal high-flow oxygen after 3 weeks.

For both twins, parenteral nutrition was started on the first day of life via an umbilical venous catheter and continued via a peripheral venous catheter. The mother’s milk was administered on their second day of life by trophic feeding through nasogastric tubes and was continued with a gradual, incremental increase in volume until full feeding was tolerated. For Twin B, oral feeding was suspended several times because of intolerance and abdominal distension. However, his serial abdominal radiographs showed nonspecific findings. The gastric tubes were eventually removed from both twins, and full oral feeding was successful upon discharge.

Their serial cranial ultrasound scans were normal. On echocardiography, Twin B had a patent ductus arteriosus with a maximum size of 2.5 mm. This was conservatively managed with fluid restriction. Follow-up echocardiography revealed a closed ductus arteriosus. His neonatal course was more complicated than his sister. He also had a right femur fracture due to osteopenia of prematurity, bilateral retinopathy of prematurity, which was treated with ranibizumab injections (Lucentis; Genentech, South San Francisco, CA), and bilateral inguinal hernias, which were treated surgically.

Four months after birth, respiratory support was weaned at the postconceptional age of 40 weeks and 5 days for Twin A and 43 weeks and 2 days for Twin B. Both twins are in excellent condition. They have returned home without respiratory or feeding support (Table 1).

**Table 1:** Clinical course at postconceptional age in the neonatal intensive care unit.

Clinical course	Twin A, girl	Twin B, boy
Extubation	28 wk and 2 d	33 wk and 3 d
Off-respiratory support	40 wk and 5 d	43 wk and 2 d
Full oral feeding	41 wk and 2 d	46 wk and 5 d
Discharge	42 wk and 1 d	47 wk and 6 d

At discharge, Twin A weighed 3708 g (47.35 centile), her length was 50 cm (16.27 centile), and her head circumference was 33.5 cm (5.21 centile). Twin B was discharged with a weight of 4120 g (3.52 centile), length of 49 cm (<1st centile), and head circumference of 34.8 cm (<1st centile). Neurological examination at the chronological age of 16 months, which corresponded to a corrected age of 12 months, demonstrated no focal deficit. Moreover, the age-appropriate neurodevelopmental-behavioral test did not exhibit any major developmental delay based on their corrected age in any of the 5 domains–motor, language, cognitive, social-emotional, and adaptive for either infant. Their growth is being monitored, and their parents are satisfied with their progress (Table 2). They are supported by appropriate allied health services and multidisciplinary medical follow-up. After discharge, Twin A had ligation of patent ductus arteriosus without complications, whereas Twin B had 1 5-day admission due to bronchiolitis. Presently, at the chronological age of 18 months (corrected age of 14 months), they are able to cruise around furniture, walk few steps without support, speak 2 syllable words, understand simple commands, and have unimpaired visual and auditory functions based on examination results.

**Table 2:** Growth parameters with prematurity-corrected percentiles at 16 months chronological age (12 months corrected age).

Growth parameter	Twin A, girl	Twin B, boy
Weight	8.6 kg (13.52centile; z score, −1.10)	8.9 kg (7.25 centile; z score, −1.46)
Length	71 cm (13.28 centile; z score, −1.11)	72.8 cm (13.9 centile; z score, −1.08)
Head circumference	43 cm (4.5 centile; z score, −1.70)	43.5 cm (1.04 centile; z score, −2.31)

## 3. Discussion

This report aimed to document the uniqueness of each patient in a premature birth, even in twins, and to emphasize that the decision to resuscitate infants born at the lower end of the viability range should be shared between clinicians and parents. Patient care should be individualized based on the risk factors and the parents’ wishes and beliefs.^[8,9]^

Infants born at periviability at 22 to 25 weeks of gestation could potentially survive with active support. However, their risk of mortality and morbidity is high.^[10]^ Nevertheless, advances in intensive care and a great willingness by obstetric and neonatal care providers to provide active care has led to a steady increase in the survival rates of extremely preterm newborns during the last decade.^[11]^

The risk of severe disability rises with increasingly extremely preterm births. Among actively managed infants, the rate of severe disability is approximately 1 in 7 at 24 weeks of gestation, 1 in 4 at 23 weeks of gestation, and 1 in 3 at 22 weeks of gestation.^[11,12]^ Patients born at 22 to 23 weeks of gestation have a higher risk of severe disability, although the data for babies born at 22 weeks are based on a small number of patients.^[2–4]^

Absolute survival and survival without severe impairment have been associated with protective factors. These include, but are not limited to, exposure to prenatal corticosteroids, female sex, singleton birth, higher birth weight, and an advanced clinical setting with experienced staff. However, the decision to administer intensive care at the lower end of viability between 22 to 23 weeks gestational age is challenging for clinicians and parents because of variable and complex ethical components and institutional practices and policies.^[12,13]^

Risk assessment and effective parental counseling, as well as an explanation of the risks and aiding in decision-making, should be undertaken. The family needs support to help them understand the expected outcomes, bearing in mind that each case is individualized. Moreover, the parents’ wishes should be considered in the decision to resuscitate.

## Acknowledgments

We are thankful to the patients and their family members for participating in this research study. The project was not funded by any organization.

## Author contributions

**Formal analysis:** Ashraf Abuobayda, Nabil Elmansoury.

**Writing – original draft:** Wafaa AlRawi.

**Writing – review & editing:** Wafaa AlRawi,TaisserAtrak, Ashraf Abuobayda, Nabil Elmansoury, Omar El Nakeib, Abhijeet Lonikar.
